# Primary gastric inflammatory myofibroblastic tumor treated with non‐exposed endoscopic wall‐inversion surgery (NEWS): A case report and literature review

**DOI:** 10.1002/deo2.243

**Published:** 2023-05-08

**Authors:** Yuka Masuda, Yoshikazu Kanazawa, Osamu Goto, Daisuke Kakinuma, Kazutoshi Higuchi, Eriko Koizumi, Ryosuke Nakata, Nobuyuki Sakurazawa, Fumihiko Ando, Mikito Suzuki, Toshiro Yoshiyuki, Shunji Kato, Ryuji Ohashi, Mitsuru Kaise, Katsuhiko Iwakiri, Hiroshi Yoshida

**Affiliations:** ^1^ Department of Gastrointestinal and Hepato‐biliary‐pancreatic Surgery Nippon Medical School Hospital Tokyo Japan; ^2^ Department of Gastroenterology Nippon Medical School Hospital Tokyo Japan; ^3^ Department of Diagnostic Pathology Nippon Medical School Hospital Tokyo Japan

**Keywords:** gastric tumor, inflammatory myofibroblastic tumor, LECS, NEWS, stomach

## Abstract

Inflammatory myofibroblastic tumor (IMT) is an intermediate malignancy with myofibroblast proliferation and inflammatory cell infiltration with malignant potential. Primary IMTs are predominantly reported in the lungs, while gastric IMTs are very rare. Therefore, no guidelines exist for the diagnosis and treatment of gastric IMTs. The present case is a 39‐year‐old man diagnosed with an asymptomatic gastric submucosal tumor. Considering the malignancy of the tumor, we selected non‐exposed endoscopic wall‐inversion surgery as the resection method and successfully performed local resection. Histopathological analysis showed myofibroblast proliferation and inflammatory cell infiltration, with a diagnosis of primary gastric IMT and negative resection margins. Immunohistochemical staining was negative for anaplastic lymphoma kinase. To the best of our knowledge, including our case, there have been 52 reported cases of primary gastric IMTs that have been treated, with several recurrent cases. In this study, we report the first case of local resection of gastric IMT using non‐exposed endoscopic wall‐inversion surgery, with a literature review.

## INTRODUCTION

An inflammatory myofibroblastic tumor (IMT) is a rare mesenchymal neoplasm with a prevalence of 0.04%–1.0% among lung tumors, first described by Brunn in 1939 as a benign pulmonary spindle cell tumor.^1^ Coffin et al. reviewed 84 cases of IMT, excluding the lungs, and reported its occurrence in various organs throughout the body. IMTs originate mostly in the abdominal soft tissues, including the mesentery and omentum, followed by the lungs, mediastinum, and gastrointestinal tract.^2^ They are characterized by myofibroblastic and fibroblastic spindle cells with infiltration of inflammatory cells.^2^, ^3^ Based on the current World Health Organization classification of soft tissue tumors, IMTs are considered intermediate tumors with a morphological spectrum between benign and malignant characteristics.^3^ Moreover, Coffin et al. suggested that pathological anaplastic lymphoma kinase (ALK) expression can be a prognostic indicator of possible local recurrence in intra‐abdominal IMT.^4^

Laparoscopic and endoscopic cooperative surgery (LECS) is a rendezvous technique using the advantages of both laparoscopy and endoscopy to minimize the resection area of the surgical specimen.^5^ Non‐exposed endoscopic wall‐inversion surgery (NEWS) is a LECS technique that prevents the exposure of the tumor surface to the abdominal cavity and facilitates precise determination of the surgical tumor margin. Thus, NEWS provides local resection with minimal excision volume.^6^, ^7^, ^8^ Therefore, in this study, we report a case of a very rare gastric IMT successfully diagnosed and treated using NEWS.

## CASE REPORT

A 37‐year‐old man underwent esophagogastroduodenoscopy screening, incidentally revealing a gastric submucosal tumor. Two years later, the patient was referred to our institution for diagnosis.

**FIGURE 1:**
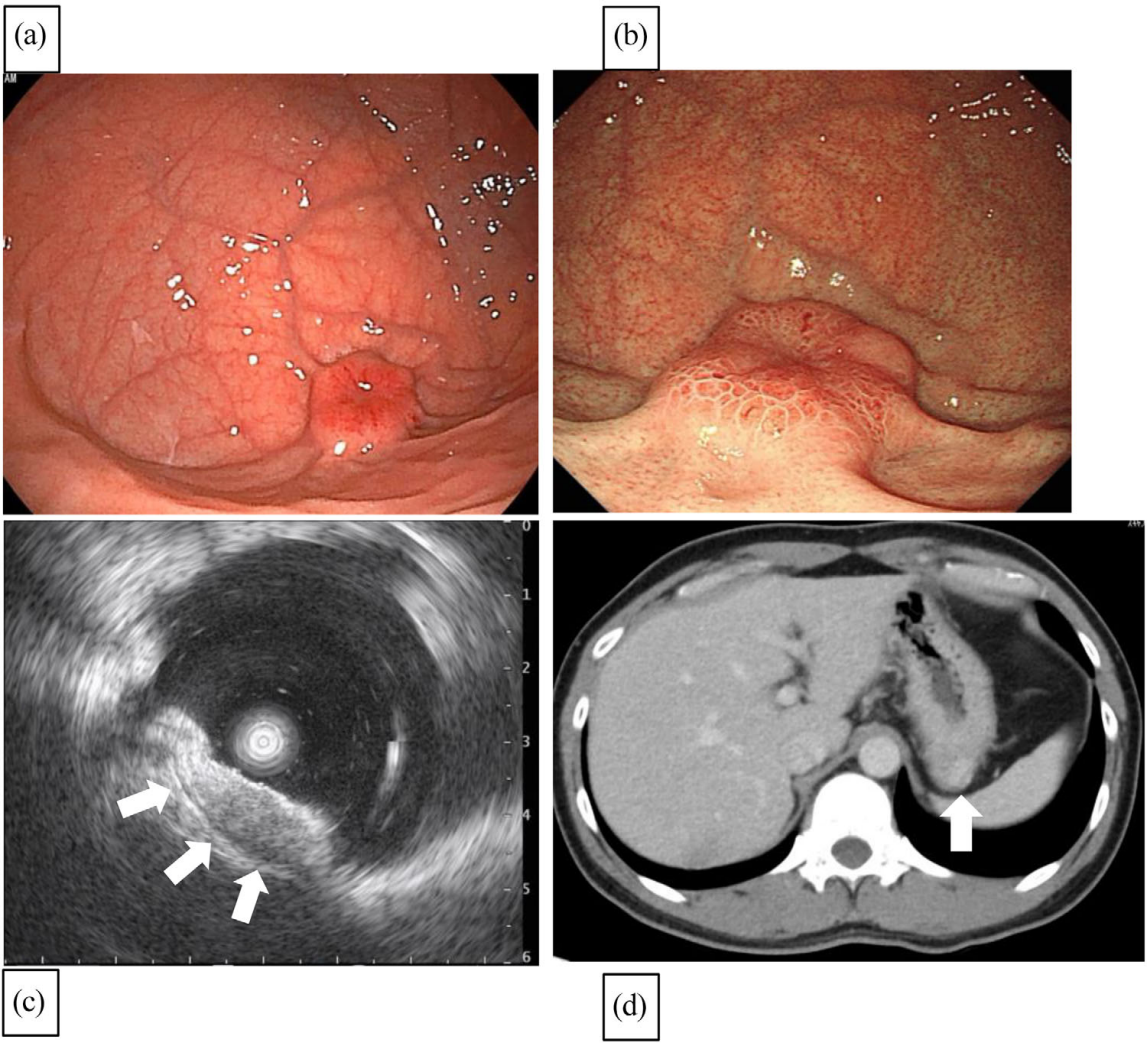
Esophagogastroduodenoscopy (EGD) and endoscopic ultrasonography (EUS) findings: (a) Previous physician's EGD findings reported a submucosal tumor‐like lesion in the fornix of the stomach. (b) EGD findings at our institution showed that the tumor was approximately 2 cm in length, and had increased in its thickness, with a reddish mucosal surface and central depression. (c) EUS revealed a tumor with a maximum diameter of approximately 20 mm with hypovascularity in the fourth layer and partially heterogeneous internal structures (white arrow). (d) Abdominal computed tomography showed no lymph nodes or distant metastases, just a tumor with a contrast effect in the same region of the gastric wall noted at endoscopy (white arrow).

Laboratory findings and tumor markers were within normal levels (cancer embryonic antigen 1.1 ng/ml, carbohydrate antigen 19‐9 < 2.0 U/ml). Esophagogastroduodenoscopy findings from the previous physician reported a lesion in the fornix, which was reported to be less than approximately 2 cm (Figure 1a). Our institution showed that the tumor was approximately 2 cm long and had increased in thickness, with a reddish mucosal surface and central depression (Figure 1b). Narrow‐band imaging with magnifying endoscopy did not reveal any cancerous characteristics of the mucosa. Endoscopic ultrasonography revealed a 1.9 cm lesion with poor vascularity in the fourth layer of the gastric wall (Figure 1c). Computed tomography showed no lymph nodes or metastases (Figure 1d). A boring biopsy of the tumor provided a specimen positive for α‐smooth muscle actin (SMA) in the proliferating spindle‐shaped cells. Therefore, the lesion was diagnosed as a leiomyoma, but surgery was performed for a reliable diagnosis for en bloc resection.

**FIGURE 2:**
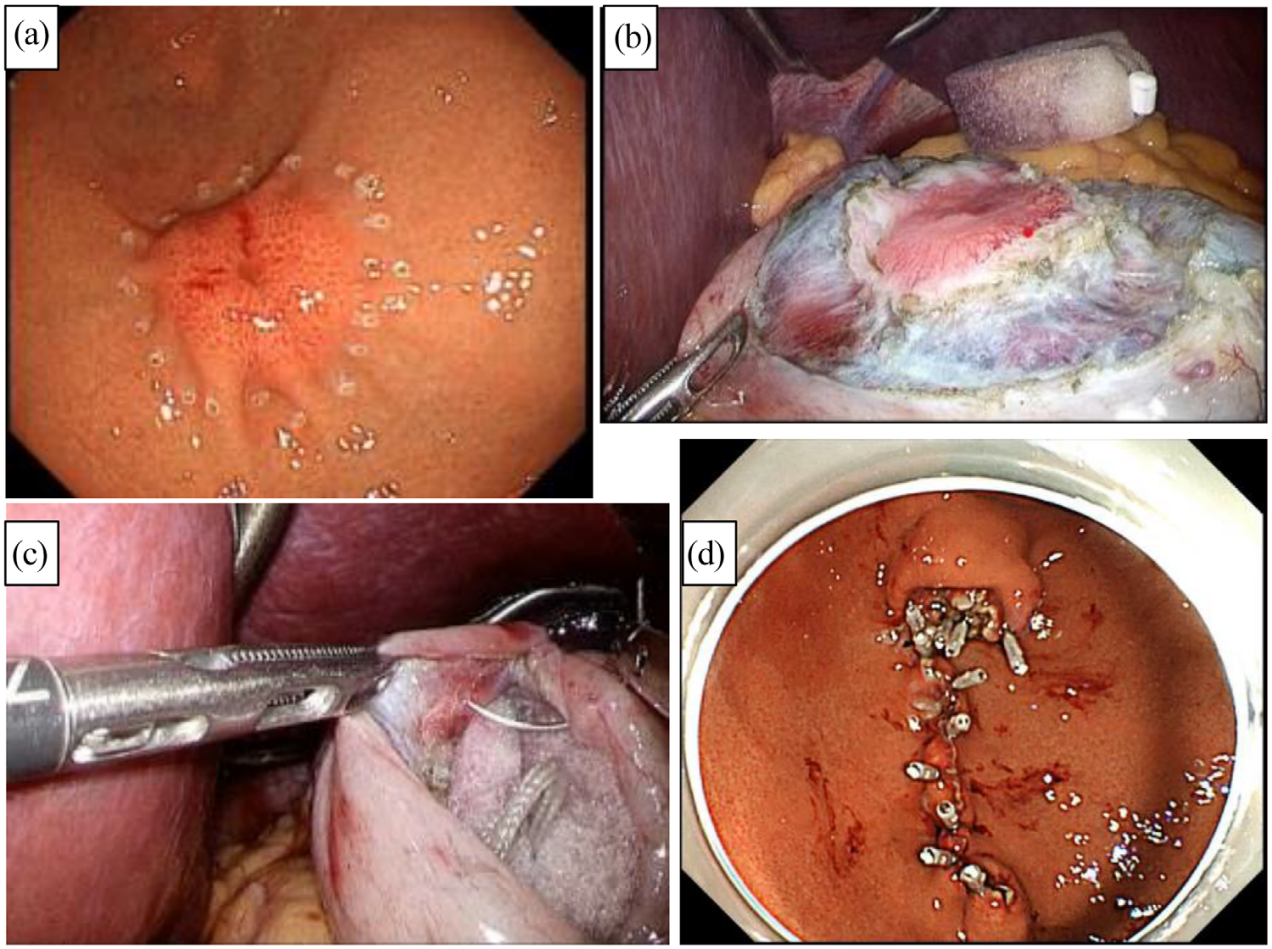
Non‐exposed endoscopic wall‐invasion technique: (a) The tumor was observed endoscopically and the margin was determined. (b) The sero‐musclar layer around the tumor was incised laparoscopically to the submucosa where hyaluronic acid solution colored with indigocarmine was endoscopically injected beforehand. (c) During laparoscopic sero‐muscular suturing, the tumor was inverted into the intragastric cavity with a sponge spacer from the external wall of the stomach. (d) The tumor was endoscopically removed with a submucosal dissection technique and retrieved transorally. The mucosal defect was closed with clipping.

Intra‐abdominal observations revealed that the tumor was present on the greater curvature/anterior wall of the fornix and had angiogenesis on the serosa. During partial resection of the stomach using the NEWS method^7^ (Figure 2a–d), the tumor margins were identified. The resection line was accurately determined by placing markings both endoscopically and laparoscopically. The tumor was patently inverted into the gastric lumen, followed by an en‐bloc resection and transoral retrieval. The operative time was 229 min, including endoscopic observation time. The patient was discharged from the hospital on postoperative day 7 without complications, and no evidence of local recurrence or distant metastasis was observed four years postoperatively.

**FIGURE 3:**
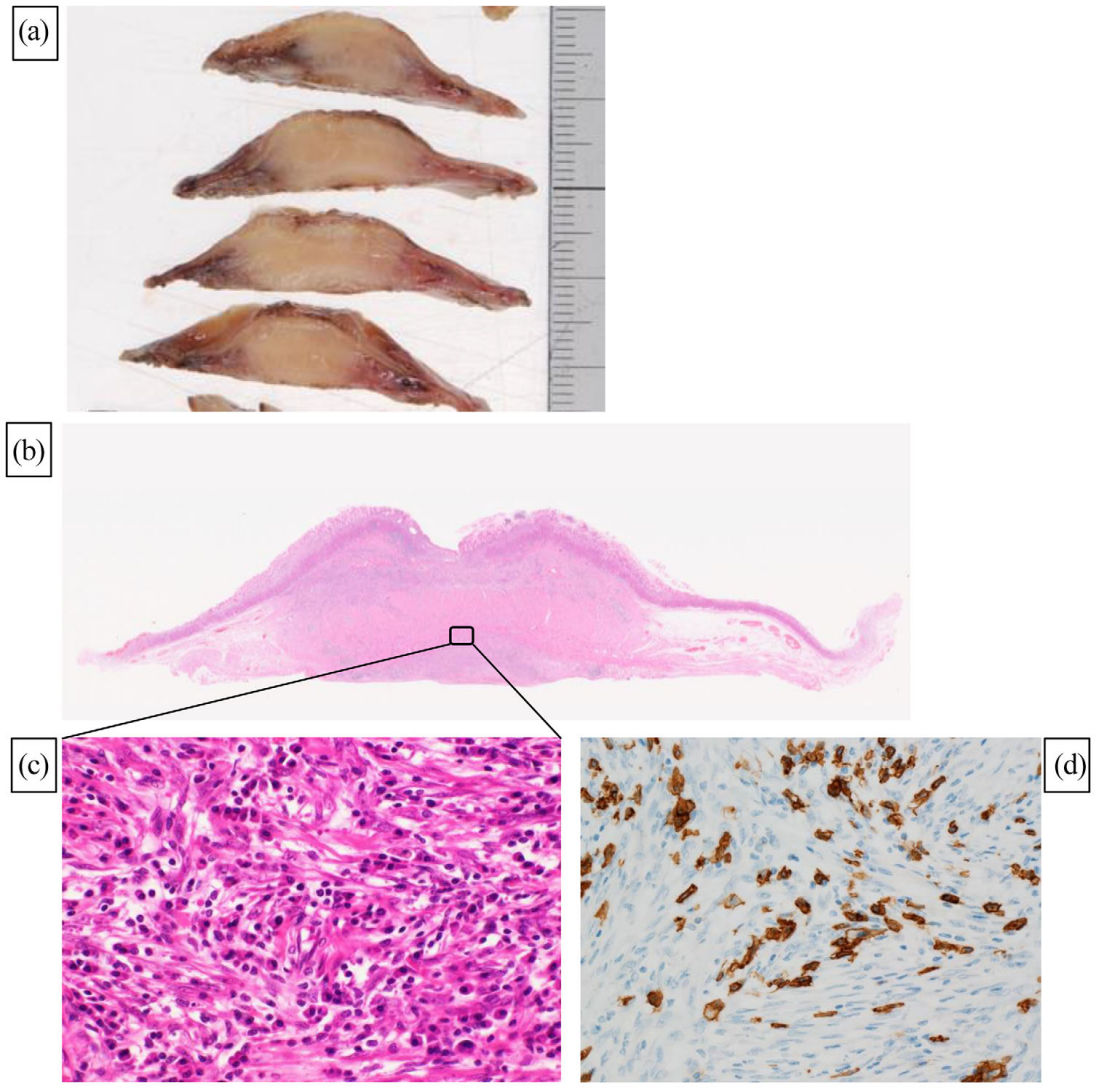
The resected specimen: (a) The tumor was 25 × 22 mm with sufficient margins of normal gastric wall tissue. The tumor cross‐section was white and homogeneous, with no internal necrotic tissue. (b) Hematoxylin‐stained loupe image showed a tumor with a mixture of cord‐like spindle‐shaped cells and inflammatory cells, with fully negative margins. (c) In the tumor, there was proliferation of spindle‐shaped myogenic cells and fibroblasts, and infiltration of inflammatory cells such as plasma cells of similar round shape. (d) Immunohistochemical staining was negative for anaplastic lymphoma kinase 1.

Macroscopically, the tumor was 2.5 × 2.2 cm in diameter, with a sufficient normal gastric wall tissue margin (Figure 3a). Pathologically, the tumor had an infiltrative growth pattern with spindle‐shaped cells growing in a cord‐like pattern and round cells with basophilic cytoplasm (Figure 3b,c). Immunohistochemical staining revealed spindle‐shaped cells positive for SMA and negative for c‐kit, whereas the round cells, positive for CD38 and CD138, were identified as plasma cells. Thus, the final diagnosis was IMT, and the tumor margins were negative. Moreover, ALK expression was negative (Figure 2d).

## DISCUSSION

IMTs are rare mesenchymal neoplasms, and some IMTs may exhibit malignant properties, including local recurrence and metastasis. Therefore, IMT is classified as a low‐grade malignancy by The World Health Organization soft tissue tumor pathology and genetic classification 2014.^2^, ^3^

Pathologically, IMT is characterized by myofibroblastic and fibroblastic spindle cells with infiltration of inflammatory cells, including lymphocytes and plasma cells.^2^, ^3^ In 1995, Coffin et al. reviewed 84 cases of extrapulmonary IMT and reported that it was more common in children and young adults, and women. The report also identified that IMTs were most prevalent in abdominal soft tissues, followed by the lung and gastrointestinal tracts. Moreover, 13 of the 53 patients (24.5%) were reported to have a recurrence within 2 years of treatment.^2^ ALK expression was associated with young‐onset and local recurrence in 59 cases of IMT with a soft tissue primary.^4^

This study also included a literature review. The terms used for the search were “IMT” with “stomach” or gastric using PubMed ((Inflammatory myofibroblastic tumor) AND (gastric) OR (stomach) ‐ Search Results ‐ PubMed (nih.gov)). As a result, 51 case reports were retrieved from the English literature, including 38 reports since 1994, and this case is based on these reports. The patients’ characteristics and treatments are presented in Table 1. The median age of the participants was 37 years (range; 0–88), and 42.3% of the patients were younger than 34 years. The median tumor size was 5.5 (1.5–22.0) cm. Additionally, 46.2% of cases had tumors ≥5 cm in size, presenting with symptoms including abdominal pain and mass palpation. In those cases, tumor tissue was confirmed as IMT before treatment in only one case, which was a bulky tumor. The surgical methods were open gastrectomy in 38 cases (73.1%) and partial resection in 28 cases (63.6%). This report is the first IMT resection by NEWS as LECS. Four patients had a recurrence, but there was limited information on the association between ALK expression and recurrence.

**TABLE 1:** Characteristics and procedures in 52 reported cases of the gastric inflammatory myofibroblastic tumor

**Characteristics and procedure**	***N* (%)**
Age	
≤34/>35 years old	22 (42.3%)/30 (57.0%)
Sex	
Male/female	18 (34.6%)/34 (65.4%)
Three portions of the stomach	
Upper/middle/lower‐third	21 (40.4%)/13 (25.0%)/18 (34.6%)
Tumor size	
≤5/>5 cm	24 (46.2%)/28 (53.8%)
Treatment approach	
EMR or ESD/LECS/laparoscopic/laparotomy/unknown	2 (3.8%)/2 (3.8%)/4 (7.7%)/38 (73.1%)/6 (11.5%)
Methods of gastrectomy method by LECS, laparoscopy, or laparotomy	
Total/distal/proximal/partial/other	3 (6.8%)/11 (25.0%)/1 (2.3%)/28 (63.6%)/1 (2.3%)
Combined resection of the other organs	
No/yes/unknown	37 (71.2%)/9 (17.3%)/6 (11.5%)
ALK expression	
Negative/positive/unknown	15 (28.8%)/20 (38.5%)/17 (32.7%)
Recurrence	
No/yes/unknown	43 (82.7%)/4 (7.7%)/5 (9.6%)

Abbreviations: ALK, anaplastic lymphoma kinase; EMR, endoscopic mucosal resection; ESD, endoscopic submucosal dissection; LECS, laparoscopic and endoscopic cooperative surgery.

In this case, preoperative endoscopic examination revealed inflammatory changes on the surface of the tumor. No definite evidence of malignancy was observed, but the possibility of malignancy was not eliminated based on its central depression, generally observed in cancers or gastrointestinal stromal tumors. Often, endoscopic biopsies do not provide sufficient tissue samples for immunohistochemical staining. The boring biopsy sample collected in this case may be composed of only the muscularis mucosa of the lesion surface. Negative oncological outcomes must be considered in complete tumor resection, including iatrogenic dissemination and incomplete resection. Therefore, this study used LECS, a rendezvous technique that minimizes the volume of tissue resected by using the advantages of laparoscopy and endoscopy. In the LECS procedures, NEWS and CLEAN‐NET, a combination of laparoscopic and endoscopic approaches for neoplasia with non‐exposure technique, do not involve transmural communication and exposure of the tumor surface to the peritoneum.^5^ Moreover, NEWS facilitates precise tumor resection by determining the area both endoscopically and laparoscopically.^6^, ^7^ Fujishiro et al. reviewed data on cases treated using NEWS and reported that it facilitates the resection of a minimum volume of tumor needed for a negative tumor margin in the stomach.^8^ Hayashi et al. reported the treatment of gastric IMT with a tumor growing extraluminally using CLEAN‐NET.^9^ There were three reasons for the selection of NEWS as a surgical procedure for this case. First, where the tumor is malignant, we considered avoiding intraperitoneal exposure during resection. Second, the tumor is less than 30 mm in diameter and can be collected orally without an abdominal cavity. Third, Classical LECS does not completely prevent exposure of gastric contents, and inverted LECS and other non‐exposure methods may be acceptable, but this case is a tumor of the anterior gastric wall, and we are well‐versed in NEWS. We previously reported a rare case of submucosal tumor in a collision tumor with a mixed adenocarcinoma and gastrointestinal stromal tumors.^10^ This case was successfully diagnosed and treated with NEWS, but according to 51 cases of gastric IMT, metastasis, and recurrence are rare, but this case should continue to be followed up.

In summary, we report a rare case of a submucosal tumor‐like gastric tumor covered with inflammatory mucosa, which was finally diagnosed as IMT. NEWS is a useful and oncologically safe technique for diagnosing and treating tumors with potential malignancy and tumor cell seeding.

## CONFLICT OF INTEREST STATEMENT

None.

## INFORMED CONSENT

The patients provided informed consent for the publication of their clinical details.
